# Supplementary material for: Exploring gestational age, and birth weight assessment in Thatta district, Sindh, Pakistan: Healthcare providers’ knowledge, practices, perceived barriers, and the potential of a mobile app for identifying preterm and low birth weight
Source: PLoS One. 2024 Apr 11;19(4):e0299395. doi: 10.1371/journal.pone.0299395 (PMC11008874; doi:10.1371/journal.pone.0299395)
Supplement: S1 File — (DOCX) [file pone.0299395.s002.docx]

**Exploring Gestational Age, and Birth Weight Assessment in Thatta District, Sindh, Pakistan: Healthcare Providers' Knowledge, Practices, Perceived Barriers, and the Potential of a Mobile App for Identifying Preterm and Low Birth Weight**

**Appendix 1**

# Semi-structured interview guide for in-depth interviews (IDI) with health care providers

# In your opinion, why it is important to determine GA?

- What methods do you use to assess GA when a woman visits this facility?
- What, in your opinion, are some of the barriers that can influence your preferred method of GA?
- How do you ascertain whether a baby was born prematurely in your practice?
- How will you define preterm birth or low birth weight, based on your experience?
- In your practice, how do you routinely manage a preterm baby or low birth weight who is born or referred to your facility?
- What are the barriers you face in your practice when referring to a preterm or low birth-weight baby?

**Table 2: Characteristics of study participants**

| **Health Care providers (n-15)** | |
| --- | --- |
| Median age (Range) | 38 (Range 22 - 59) years |
| Gender  -Female  -Male | 13 (86.7%)  2 (13.3%) |
| Medical cadre |  |
| -Obstetrician | 3 (20.0%) |
| -Doctors | 3 (20.0%) |
| -Community mid-wives | 5 (33.3%) |
| -Nurses | 2 (13.3%) |
| -Lady health visitors | 2 (13.3%) |
| Years of experience  Median (Range) | 12.1 (1-26 years) |
| **Participants of focus group discussions (n-30)** | |
| Median age (Range) | 30 (19-39) years |
| Educated status |  |
| -Uneducated | 26 (86.7%) |
| -Educated | 4 (33.3%) |
| Occupation |  |
| -Housewife | 28 (93.3%) |
| -Teacher | 2 (6.7%) |
