# Supplementary material for: Exploring gestational age, and birth weight assessment in Thatta district, Sindh, Pakistan: Healthcare providers’ knowledge, practices, perceived barriers, and the potential of a mobile app for identifying preterm and low birth weight
Source: PLoS One. 2024 Apr 11;19(4):e0299395. doi: 10.1371/journal.pone.0299395 (PMC11008874; doi:10.1371/journal.pone.0299395)
Supplement: Attachment — Submitted filename: Responses to reviewers comments_23-12-23.docx [file pone.0299395.s003.docx]

**PONE-D-23-24083**

**“Exploring gestational age, and birth weight assessment in Thatta district, Sindh, Pakistan: healthcare providers' knowledge, practices, perceived barriers, and the potential of a mobile app for identifying preterm and low birth weight.”**

**The point-by-point responses to reviewers’ comments**

On behalf of all authors, I express my sincere gratitude for your thorough review of our research paper, and we truly appreciate the time and expertise you dedicated to the process. Your contributions are invaluable, and we are grateful for your commitment to advancing academic excellence.

We've made revisions using the "Track Changes" function in the MS Word document, clearly marked as such.

Additionally, we've included a clean copy following the acceptance of track changes.

| **Reviewer's comments** | **Author's response(s)** |
| --- | --- |
| Reviewer #1: Simple and well-written article. Good sample size within depth interview quotes are well defined considering qualitative study. No grammatical errors. The methodology looks fine. I did not find any major issues in the study conduct. | Thank you for the comments. |
| Reviewer #2: The study is well written. However, the authors should describe the type of mobile app that was used to explore its potential in the current study. e.g. is it a standard mobile app, its name, was this app created for this study, what information is required in it to get the desired output, etc. | Thank you for your comment.  In our study, we did not utilize a specific mobile app to explore its potential. We explored the opinions of health providers on a mobile phone application for the identification of preterm and low birth weight.  However, in the future, we plan to conduct a quantitative study to develop a regression predictive model. Based on this model we will develop an app. This app will use post-natal foot length as an input. |

**Journal’s requirement:**

1. Please ensure that your manuscript meets PLOS ONE’s style requirements, including those for file naming.

**Response**: We carefully review the journal’s guidelines and revise the manuscript.

2. Please include a complete copy of PLOS’ questionnaire on inclusivity in global research in your revised manuscript. Our policy for research in this area aims to improve transparency in the reporting of research performed outside of researchers’ own country or community. The policy applies to researchers who have travelled to a different country to conduct research, research with Indigenous populations or their lands, and research on cultural artefacts. The questionnaire can also be requested at the journal’s discretion for any other submissions, even if these conditions are not met. Please find more information on the policy and a link to download a blank copy of the questionnaire here: https://journals.plos.org/plosone/s/best-practices-in-research-reporting. Please upload a completed version of your questionnaire as Supporting Information when you resubmit your manuscript.

**Response:** The study was conducted in the rural district of Thatta, by the primary author who is a citizen of this country. The primary author did not travel from another country to collect the data. Therefore, we believe this is not applicable in our case.

3. We suggest you thoroughly copyedit your manuscript for language usage, spelling, and grammar. If you do not know anyone who can help you do this, you may wish to consider employing a professional scientific editing service.

**Response:** One of the authors is well-versed in English and reviewed this manuscript for language usage, spelling, and grammar.

4. In your Data Availability statement, you have not specified where the minimal data set underlying the results described in your manuscript can be found. PLOS defines a study's minimal data set as the underlying data used to reach the conclusions drawn in the manuscript and any additional data required to replicate the reported study findings in their entirety. All PLOS journals require that the minimal data set be made fully available.

**Response:** Since this was a qualitative study, we have added the interview guides.

5. PLOS requires an ORCID iD for the corresponding author in Editorial Manager on papers submitted after December 6th, 2016. Please ensure that you have an ORCID iD and that it is validated in Editorial Manager. To do this, go to ‘Update my Information’ (in the upper left-hand corner of the main menu), and click on the Fetch/Validate link next to the ORCID field. This will take you to the ORCID site and allow you to create a new iD or authenticate a pre-existing iD in Editorial Manager. Please see the following video for instructions on linking an ORCID iD to your Editorial Manager account: <https://www.youtube.com/watch?v=_xcclfuvtxQ>

**Response:** We carefully checked and added the ORCID ID of the corresponding author.

6. Please review your reference list to ensure that it is complete and correct. If you have cited papers that have been retracted, please include the rationale for doing so in the manuscript text, or remove these references and replace them with relevant current references. Any changes to the reference list should be mentioned in the rebuttal letter that accompanies your revised manuscript. If you need to cite a retracted article, indicate the article’s retracted status in the References list and also include a citation and full reference for the retraction notice.

**Response:** We have carefully checked and corrected the references according to journal’s recommendations.
